# A Psychoeducational Support Group Intervention for People Who Have Attempted Suicide: An Open Trial with Promising Preliminary Findings

**DOI:** 10.1007/s10597-022-00978-y

**Published:** 2022-05-30

**Authors:** Myfanwy Maple, Sarah Wayland, Tania Pearce, Rebecca Sanford, Navjot Bhullar

**Affiliations:** 1 Faculty of Medicine and Health, University of New England, Armidale, Australia; 2 School of Social Work and Human Service, Thompson Rivers University, Kamloops, Canada; 3 Discipline of Psychology, Edith Cowan University, Joondalup, Australia

**Keywords:** Suicide, Suicide attempt, After care, Support group, Psychoeducational intervention

## Abstract

Psychoeducational groups have been used to address many health needs. Yet, there are few such options available for people who have attempted suicide. This study presents preliminary findings from an open trial of Eclipse, an 8-week closed, psychoeducational group for people who have attempted suicide. It examined the effectiveness of the Eclipse program in reducing suicidal ideation, depressive symptoms, perceived burdensomeness and thwarted belongingness, and increasing resilience and help-seeking. Results showed statistically significant improvements in depressive symptoms, perceived burdensomeness, resilience and help-seeking from baseline (T1) to immediate post-test (T2), and in perceived burdensomeness from T1 to 1-month follow-up (T3). A pervasiveness analysis showed that over half of the participants reported improvements in key study outcomes, respectively, as a result of participating in the Eclipse group. Psychoeducational support groups could provide broad application for those who have previously attempted suicide in decreasing severity of suicidal thinking by reductions in depressive symptoms, burdensomeness, and thwarted belongingness.

**Supplementary Information:**

The online version contains supplementary material available at 10.1007/s10597-022-00978-y.

## Introduction

In the context of unprecedented funding and activity in suicide prevention activities in Australia, suicide remains a leading cause of death. The Australian Bureau of Statistics (ABS, 2021) reported 3318 recorded as suicide, with a preliminary death rate of 12.9 per 100,000. Yet, predicting for whom, and when, suicide may occur remains only slightly better than chance (Franklin et al., 2017). While difficult to accurately calculate the prevalence of suicide attempt and suicidal ideation, some reports indicate that there are 20 to 30 suicide attempts for each suicide death (World Health Organization, WHO, 2014) and that ideation and attempt may be increasing across the population (Franklin et al., 2017; Nock et al., 2008).

Prior history of suicide attempt is a strong predictor of future suicide death, and risk of suicide is highest within close proximity to discharge from hospital. A systematic review of studies examining the rates of suicide after discharge from psychiatric facilities reported 484 suicides per 100,000 person-years using a pooled estimate of post discharge suicide deaths (Chung et al., 2017). The provision of evidence-based aftercare services can reduce this short-term risk, and more services are becoming available (Simon et al., 2018), often focused on the immediate post-discharge period. Although suicide rates were highest within 3 months after discharge and among patients admitted with suicidal ideation or behaviors, suicide risk remains high for many years (Chung et al., 2017). This can put the onus on family members or close friends to provide informal care and support to the person following a suicide attempt, thus exacerbating caregiver burden and suicide risk for carers (Maple et al., 2021). Research has suggested the development of adequate supports for carers to mitigate their future suicide risk (Bhullar et al., 2021). However, there are few, non-clinical supports available for people with prior suicide attempts who may continue to live with persistent suicidal ideation and repeated attempts. Yet, such supports are reported as being desired by those who have these experiences (Hom et al., 2021). One option may be to provide support to suicide attempt survivors via a psychoeducational support group in a community setting.

Support groups are a non-clinical adjunct to clinical services. Psychoeducation, as defined by Vreeland (2012, p. 50), is an ‘evidence-based psychotherapeutic intervention’ that aims to teach or educate individuals and/or their families. The psychoeducation may primarily focus on the nature of the illness or condition they have been diagnosed with, how to live with the condition and strategies or skills that may assist with recovery and living well. Such support groups are organized from those professionally led by a trained facilitator to informal groups managed by peer leaders (Feigelman et al., 2008), with joint facilitation by both professional and peer facilitators also being reported (Hom et al., 2018; Lara-Cabrera et al., 2016). These types of interventions have been found to be highly effective when focused on addressing a single problem or issue (Tyler et al., 2019).

Although psychoeducation is a recognized and important evidence-based intervention often used in the context of support and peer groups (Barber et al., 2008; Castle et al., 2010; Hom et al., 2018; Poole et al., 2015), the specific role of these groups in improving mental health outcomes of participants is rarely examined. In instances where these outcomes are reported show promising results. For example, a meta-analysis of seven randomized controlled trials with a total of 849 participants comparing peer support interventions with usual care found a significantly greater reduction than usual care (− 0.59, 95% CI, − 0.98, − 0.21; p = 0.002), a findings that was comparable to cognitive behavioral therapy outcomes (Pfeiffer et al., 2011).

To our knowledge only one study examining the effectiveness of support groups in reducing suicidal ideation in suicide attempt survivors has been published to date. This is an open trial reported by Hom et al. (2018) on preliminary findings of the impact of the Survivors of Suicide Attempt (SOSA) support group, run by Didi Hirsch Mental Health Services (2014) in the US, for people who have previously attempted suicide. The SOSA program is an 8-week psychoeducation program, run jointly by professional and peer facilitators dyads. The evaluation (Hom et al., 2018) found participation by those with a history of suicide attempts (*n* = 92), in a closed group over an 8-week period, resulted in significant reductions in suicidal symptoms (hopelessness, suicidal ideation, suicidal desire, and suicidal intent), and increased resilience. However, these authors reported only modest effects of the SOSA program in reducing suicidal symptoms. The authors also noted that continued “engagement of suicide attempt survivors into these treatment modalities may be beneficial, to markedly impact suicide rates, exploration of alternate suicide prevention approaches among attempt survivors” (Hom et al., 2018, p. 290). The authors concluded that the SOSA intervention was cost effective and accessible for individuals by jointly addressing the attempting behaviors whilst also enhancing “social connection and resilience while also overcoming stigma-related barriers” (Hom et al., 2018, p. 303).

The current study reports on the preliminary findings from the Eclipse group, which is an Australian version of the Didi Hirsch’s SOSA group (Didi Hirsch Mental Health Services, 2014), the focus of the Hom et al. (2018) evaluation. The Eclipse group was adapted for the Australian context (including Australian-based activities and referral pathways) and the evaluation expanded to measure outcomes beyond immediate post-test. The aim of the present study was to provide preliminary findings of the evaluation of the Eclipse program in reducing suicidal ideation as a primary outcome and decreasing depressive symptoms, perceived burdensomeness and thwarted belongingness, and increasing resilience and help-seeking as secondary outcomes.

## Method

The Eclipse program is facilitated through Lifeline Australia sites, initial findings from three trial locations are reported in the current study. Participants were recruited through the local community settings (e.g., general practitioners, flyers, newspaper advertisements), interested individuals were directed to make contact with, and discuss their eligibility for, Eclipse with group facilitators.

The Eclipse group was initially piloted from February 2017 through November 2017 in one Lifeline centre to ensure the original Didi Hirsch Support After Suicide group content and the evaluation design were robust for trialing with a broader sample at multiple Australian sites. The recommendations arising from the pilot included replacing the existing paper-based suicide safety planning tool with an Australian mobile application for suicide safety planning (Melvin & Gresham, 2016) and the inclusion of The Suicidal Ideation Attributes Scale (SIDAS; van Spijker et al., 2015) following difficulties with the informal suicide assessment used in the pilot location and to ensure a validated tool was being used for the primary outcome measure. The open trial of the Eclipse program ran from January 2018 to December 2019 at a further two Lifeline centres, with follow-up from these groups occurring in May 2020.

Ethics approval was granted by the University of New England Human Research Ethics Committee (HE16-195). All participants provide written consent. This research protocol (Maple et al., 2020) was retrospectively registered with the Australian New Zealand Clinical Trials Registry (ANZCTR) (ACTRN:12619000542190) on 05/04/2019: https://www.anzctr.org.au/Trial/Registration/TrialReview.aspx?id=377318&isReview=true. All authors accept responsibility for the manuscript and declare there are no known conflicts of interest.

### Participants

Those eligible to participate in the intervention were aged 18 years or over at commencement of the group and reported a history of suicide attempt and sound understanding of the English language, and were able to attend the physical location of the group and were not actively suicidal. Prior to participation in the group, interested participants undertook an intake and screening interview with the professional group facilitator who assessed the participant’s suitability against the eligibility criteria. The key eligibility criteria was past suicide attempt, which was asked as a yes/no question. Only those with self-reported prior suicide attempt continued with eligibility screening. Current suicidality was also assessed through a narrative discussion between the professional facilitator and potential participant. If a potential participant was in immediate suicide risk, they were referred to clinical services and were not eligible for the support group at that time, given the non-clinical, psychoeducational focus. When a participant was accepted to participate in the group, the facilitator informed them of the research associated with the group which was undertaken by the authors, provided them with the information about the research, and the consent information. Participants were clearly informed that they could opt into or out of the research, and that this would not influence their participation in the support group. Participants were further informed that only those participants who agreed to participate in the research component would have their de-identified information made available to the research team. All trial group participants agreed to participate in the research.

### Intervention

The program was delivered in accordance with the intervention prescribed by the US model developed by the Didi Hirsch Centre (Didi Hirsch Mental Health Services, 2014) (with the aforementioned adaptations) over an 8-week period. The Eclipse psychoeducational support group topics and group processes are described in the Supplementary File 1.

### Data Collection Procedure

Data collection was embedded within the implementation of the intervention in accordance with the principles of co-creation (Pearce et al., 2020). To achieve this, the facilitators simultaneously administered the collection of quantitative data and the delivery of the program during the 8-week scheduled group delivery. Author (SW) monitored the data collection process and proactively maintained communication with the facilitators to ensure data were collected at each appropriate time point, and to discuss any issues related to data quality or participant attrition. As illustrated in Table 1, measurements were taken by the facilitator at baseline prior to commencing the group content in Week 1 (T1), immediately after the final group delivery in Week 8 (immediate post-test, T2), and by the researchers at 1-month follow-up (T3).

**Table 1:** Data collection timepoints

T1^a^	T2^a^	T3^a,b^
Baseline (Prior to Week 1)	Immediate post-test (Week 8)	1-month Follow-up
Information for participants Consent Demographics (age, gender, prior attempt/s) PHQ-9 INQ-15 RAS SIDAS	PHQ-9 INQ-15 RAS SIDAS	PHQ-9 INQ-15 RAS SIDAS

*PHQ-9* Patient Health Questionnaire-9, *INQ-15* Interpersonal Needs Questionnaire-15, *RAS* Resilience Appraisals Scale, *SIDAS* Suicidal Ideation Attributes Scale

^a^Data collected by Lifeline group facilitators

^b^Data collected by researchers

### Measures

Key demographic variables (e.g., age, sex, suicide attempt history) were collected prior to the start of Week 1 (T1). In addition to the demographic variables, the following psychological measures were used: Cronbach’s alphas (α), obtained in the current study, are reported in Table 2.

**Table 2:** Means, standard deviations (SD), bivariate correlations, and Cronbach’s αs of key study variables at baseline (T1)

Variables	1	2	3	4	5	6	7	8	9	10
1. Depressive symptoms	–	.40*	.44*	− .20	− .26	− .15	< − .07	.55**	.05	.13
2. Perceived burdensomeness		–	.61***	− .15	− .24	− .13	.03	.79***	− .30	.35
3. Thwarted belongingness			–	− .46*	− .51**	− .47*	− .10	.47**	− .24	.11
4. Resilience (total)				–	.86***	.87***	.66***	− .11	.24	.03
5. Emotion coping					–	.75***	.27	− .23	.35	− .13
6. Situation coping						–	.33	− .13	.15	− .02
7. Social Support							–	.10	.07	.24
8. Suicidal ideation								–	− .25	.29
9. Age									–	− .28
10. Sex										

*n*	32	40	40	40	40	40	40	32	–	–
Mean (*SD*)	15.84 (6.15)	3.77 (1.85)	4.49 (1.31)	32.93 (10.00)	9.90 (4.56)	11.60 (3.88)	11.43 (4.64)	20.81 (13.63)	–	–
Cronbach’s α	.84	.92	.85	.87	.88	.86	.85	.81	–	–

*n* = 29 for correlations with listwise deletion. Depressive symptoms assessed by PHQ-9; Perceived burdensomeness and thwarted belongingness assessed by INQ-15; Resilience (total) and Emotion coping, Situation Coping and Social Support subscales assessed by RAS; and Suicidal Ideation assessed by SIDAS. Sex: Male = 1; Female = 2

*p < .05, **p < .01, ***p < .001

#### Depressive Symptoms

The Patient Health Questionnaire (PHQ-9; Kroenke et al., 2001), a 9-item self-report measure, is used to assesses the severity of depressive symptoms. Using a 4-point Likert scale ranging from 0 (not at all) to 3 (nearly every day), respondents are asked to rate the frequency of depression symptoms in the last 2 weeks. Items are summed to create a composite score, with higher scores reflecting greater symptom severity.

#### Burdensomeness and Thwarted Belongingness

The Interpersonal Needs Questionnaire (INQ-15; Van Orden et al., 2012), a 15-item measure, is used to assess beliefs about the extent to which individuals feel like a burden on other people in their lives (i.e., perceived burdensomeness: 6 items) and the extent to which they feel connected to others (i.e., thwarted belongingness: 9 items including 6 reverse-scored items). Items are rated on 7-point Likert scale ranging from 1 (not at all true for me) to 7 (very true for me). Items are averaged to create composite scores for the two subscales.

#### Resilience

The Resilience Appraisals Scale (RAS; Johnson et al., 2010), a 12-item self-report measure, was used to assess respondents’ appraisal of their ability to cope with emotions, solve problems, and seek social support. Items are rated on a 5-point Likert scale ranging from 1 (strongly disagree) to 5 (strongly agree), with higher scores indicating greater resilience. Items are summed to create composite scores for the three subscales and the overall score.

#### Suicidal Ideation

The Suicidal Ideation Attributes Scale (SIDAS; van Spijker et al., 2014) was used to assess severity of suicidal thoughts. The SIDAS comprises five items, each assessing a specific attribute of suicidal thoughts (i.e., frequency, controllability, closeness to attempt, level of distress associated with the thoughts, and impact on daily functioning) over the past month. Items are rated on an 11-point Likert scale ranging from 0 (never/not close at all/not at all) to 10 (always/full control/made an attempt/extremely). Items are summed to create a composite SIDAS score.

### Data Analytical Plan

We first conducted bivariate correlations (using Pearson’s *r*) among all key study variables at T1. For the main analyses, we conducted paired samples *t*-tests to examine the effectiveness of the support program in terms of self-reported changes in depressive symptoms, perceived burdensomeness, thwarted belongingness, resilience, and suicidal ideation from baseline (T1) to immediate post-test (T2), and from T1 to 1-month follow-up (T3). The decision to use a *t*-test instead of a repeated measures analyses of variance was made to detect any statistically significant effect due to very small sample sizes and different participants providing completed scores at different time points. Further, to understand the pervasiveness of the effect of the intervention (Speelman & McGann, 2020), we conducted a pervasiveness analysis to explore the performance of the majority of participants on the key outcomes as a result of participating in the Eclipse group.

## Results

Our final sample at T1 was 43 participants, who participated in any one of the nine groups that occurred across three sites during the trial period. Due to the embedded nature of the data collection which relied on group facilitators to provide guidance on data collection at T1 and T2, not all data were collected nor did all participants provide complete data at each timepoint. Researchers and facilitators were present for data collection at T3, which also included a researcher-facilitated focus group (not reported here). Two participants did not complete baseline data collection, so were excluded from all future analysis. For those providing demographic information (*n* = 33), mean age was 49.33 years (*SD* = 15.65; range = 20–76 years), and 34 participants reported their sex (women = 60.5%). Finally, 32 participants reported their prior suicide attempts (yes = 72.1%). Three participants were reported as leaving Eclipse during the 8-week program, one of whom died by suicide after formally withdrawing from the group. Different participants provided data for each measure across 3 timepoints: 32 participants provided complete data at T1, 24 participants at T2, and 17 participants at T3. The remaining participants were lost to follow-up, or data were not collected. As a limitation of this embedded design, and the extent of the missing data, we removed participants without complete data for each scale or timepoint analysis resulting in different numbers of participants across analyses.

### Descriptive Statistics

Table 2 provides a summary of descriptive statistics and bivariate correlations of key study variables at baseline (T1). As expected, participants’ reported severity of depressive symptoms was significantly associated with higher levels of perceived burdensomeness, thwarted belongingness, and suicidal ideation. Greater perceptions of burdensomeness and thwarted belongingness were also significantly associated with higher levels of suicidal ideation. Greater resilience (total score), emotion coping, and situation coping were significantly associated with lower levels of thwarted belongingness, but seeking social support was not significantly related with perceived burdensomeness and thwarted belongingness. Resilience (total score) and three subscales (emotion coping, situation coping, and social support) were not significantly associated with depressive symptoms and suicidal ideation at T1. Finally, age and sex variables were not systematically related with any of the psychological variables. Therefore, these demographic variables were not controlled for in subsequent analyses.

### Paired Samples *t*-Tests

Results from paired samples *t*-tests, summarized in Table 3, showed that depressive symptoms and perceived burdensomeness significantly decreased, and resilience (total) and seeking social support significantly increased from T1 to T2; perceived burdensomeness significantly decreased from T1 to T3, mainly representing medium to large effect sizes.

**Table 3:** Summary of paired-samples t-tests: means, standard deviations (SD), t-test values, and Cohen’s d effect sizes

Variables	T1 *M* (*SD*)	T2 *M* (*SD*)	*T* (*df*)	Cohen’s *d*	T1 *M* (*SD*)	T3 *M* (*SD*)	*T* (*df*)	Cohen’s *d*
	[*n* = 25]		**2.63 (24)** ***p* = .015**	**.53**	[*n* = 16]			
Depressive symptoms	16.32 (5.95)	13.28 (5.89)			17.38 (5.91)	16.31 (5.76)	1.08 (15) *p* = .298	.27
	[*n* = 29]		**2.88 (28)** ***p* = .007**	**.54**	[*n* = 19]		**2.21 (18)** ***p* = .040**	**.51**
Perceived burdensomeness	3.94 (1.76)	3.11 (1.73)			3.79 (1.79)	2.91 (1.44)		
	[*n* = 29]		1.87 (28) *p* = .072	.35	[*n* = 19]		1.48 (18) *p* = .156	.34
Thwarted belongingness	4.48 (1.17)	4.05 (1.27)			4.61 (1.38)	3.99 (1.34)		
	[*n* = 29]		** − 2.25 (28)** ***p* = .033**	**.42**	[*n* = 18]			
Resilience (total)	33.07 (9.55)	36.45 (9.34)			30.89 (9.35)	35.33 (10.87)	− 1.43 (17) *p* = .171	.34
Emotion Coping	10.24 (4.28)	11.31 (3.69)	− 1.55 (28) *p* = .132	.29	9.22 (3.87)	10.22 (3.62)	− .91 (17) *p* = .373	.22
Situation Coping	12.07 (3.68)	12.69 (3.96)	− .86 (28) *p* = .397	.17	10.89 (3.72)	11.89 (3.71)	− .78(17) *p* = .444	.19
Social Support	10.76 (4.91)	12.45 (3.92)	** − 2.08 (28)** ***p* = .046**	**.39**	10.78 (4.57)	13.22 (5.28)	− 1.72 (17) *p* = .104	.40
	[*n* = 22]				[*n* = 16]			
Suicidal ideation	21.59 (13.32)	18.77 (14.11)	1.00 (21) *p* = .330	.21	24.13 (13.80)	23.94 (12.23)	.08 (15) *p* = .937	.02

Baseline = T1 and Immediate post-test = T2; and 1-month follow-up = T3. Cohen’s *d*: .20 (small); .50 (medium); and .80 (large). Sample sizes and participants differ in each timepoint pair comparison. Statistically significant differences based on *t*-tests and significant effects in bold

### Analysis of Pervasiveness

We computed change scores by subtracting pre-test scores from post-test scores for all the outcome variables from T1 to T2 and from T1 to T3, with negative values of difference scores for depressive symptoms, perceived burdensomeness, thwarted belongingness, and suicidal ideation representing an improvement whereas positive difference scores for resilience (total), emotion coping, situation coping, and social support indicating an improvement. Table 4 shows the proportion of participants who showed improvement, no change, or deterioration in the outcomes from T1 to T2, and from T1 to T3, respectively. For this pilot trial, we used a criterion of 50% or greater to consider an improvement in the majority of the participants in the sample. Results showed that overall the majority of the study participants reported improvements in depressive symptoms, perceived burdensomeness, thwarted belongingness, resilience (total), and seeking social support from T1 to T2, and from T1 to T3, respectively, with an improvement in emotion coping from T1 to T2 only.

**Table 4:** Summary of pervasiveness analysis showing improvements in key study outcomes

Variables	Difference (T2 − T1)			Difference (T3 − T1)		
	% Improvement	% No change	% Deterioration	% Improvement	% No change	% Deterioration
Depressive symptoms	**76**	4	20	**50**	6.3	43.8
Perceived burdensomeness	**75.9**	6.9	17.2	**63.2**	15.8	21.1
Thwarted belongingness	**58.6**	0	41.4	**52.6**	10.5	36.8
Resilience (total)	**75.9**	0	24.1	**61.1**	11.1	27.8
Emotion coping	**58.6**	20.7	20.7	44.4	11.1	44.4
Situation coping	44.8	13.8	41.4	**94.4**	5.6	0
Social support	**65.5**	0	34.5	**66.7**	5.6	27.8
Suicidal ideation	45.5	13.6	40.9	43.8	18.8	37.5

T1 = Baseline (Prior to Week 1), T2 = immediate post-test (Week 8), T3 = 1-month follow-up

Bold values show improvements in the outcomes in ≥  50% of the participants

## Discussion

The present study examined the effectiveness of the Eclipse support group for people who have attempted suicide. Eclipse was developed to simultaneously reduce factors associated with suicide risk, while increasing resilience within a psychoeducational setting. Although our study sample was small, the preliminary results of this open trial are promising. While participants were attending the weekly groups (from T1 to T2), significant reductions were identified in depression severity and perceived burdensomeness as well as increased resilience overall, primarily driven by the change in the social support subscale of the RAS. Even with a very small sample size, which declines across the timepoints, the effect sizes ranged from medium to large. This is consistent with the literature on the role of social support as a protective factor against suicide (Kleiman & Liu, 2013; You et al., 2011). In particular, two nationally representative studies examining the relationship between social support and suicide attempt found strong evidence suggesting that social support is protective against suicide attempts (Kleiman & Liu, 2013). As a modifiable protective factor, social support presents an important opportunity for intervention (Kleiman & Liu, 2013).

There was a reduction in suicidal ideation from T1 to T2, however, this change was not statistically significant. This could be due to inadequate power to detect any significant effect in a small study sample. We observed that retention of these gains, such as significant reduction in burdensomeness was significant at T3, however, the changes in the remaining study outcomes were not statistically significant. We also examined the pervasiveness of the effect of the psychoeducational intervention to understand the level of improvements on key study outcomes in the majority of participants (Speelman & McGann, 2020). An analysis of pervasiveness indicated that ≥ 50% of the study participants reported improvements in depressive symptoms, burdensomeness, belongingness, resilience (total), and seeking social support as a result of participating in this trial Eclipse program. While our pilot sample is very small, and we report these findings with appropriate caution, they are encouraging in relation to the ongoing outcomes from participation in the Eclipse program. Future evaluation of the scaled-up Eclipse program should use the criterion of 80% or more representing the “majority” of participants reporting an improvement, therefore, demonstrating a strong effect (Speelman & McGann, 2020).

In their analysis of the US version of this group (SOSA), Hom et al. (2018) report similar findings with a reduction in suicidal ideation, hopelessness, suicidal desire, and suicidal intent, and increase in resilience, indicating that this psychoeducational group has broad appeal. The authors state, “[i]t is possible that SOSA facilitated reductions in hopelessness and suicidal symptoms by increasing participants’ sense of belonging and reducing perceptions of burdensomeness” (p. 294). Such sentiment applies to the findings reported in the current open trial. Given the emerging evidence regarding status as a suicide attempt survivor as a concealable identity (Fulginiti & Frey, 2018), there are likely many factors that influence the decision to voluntarily disclose this experience to others. In one sense, this may protect the person against stigmatizing or unhelpful responses, though it also limits the support that social networks can provide. Yet, in a safe environment where membership requires prior suicide attempt, new ways of living can be explored among others where social connection and belonging can be facilitated (Lakeman & FitzGerald, 2008). This support group offers an important space for giving and receiving social support in a non-judgmental, non-stigmatizing environment. While receiving support and understanding from others may increase feelings of belonging, providing support to others in the group may also increase feelings of purpose and helpfulness to others, thereby reducing feelings of being a burden on others, highlighting the importance of peer support (Hom et al., 2018, 2021).

The findings reported here should be interpreted with caution and in light of the limitations. The small sample size and challenges with routine data collection impact on the strength of the findings. However, with commitment to the co-creation process, and full engagement with service providers and peer facilitators, this has resulted in a lot of learning in relation to data collection and quality which is evolving in line with the evolution and further roll out of Eclipse to additional sites. As more Lifeline sites are established in Australia, program fidelity also needs to be examined to ensure the same program is being delivered to different groups and in different locations. In this trial one facilitator had been trained by Didi Hirsch to deliver the curriculum, and this individual then trained subsequent facilitators. The authors trained all facilitators in data collection procedures, and regular discussions were held between all parties to ensure consistency across sites. Fidelity in delivery will be further challenged as groups are modified and some move into a secure online environment to address social distancing requirements imposed by the current COVID-19 pandemic.

## Conclusion

Supporting those who have survived prior attempts to end their lives is a vital suicide prevention activity. Psychoeducational support groups, such as the Eclipse have a role in the delivery of support to people who have attempted suicide. Our preliminary findings (from T1 to T2) indicate that the Eclipse support group helps in reducing depression severity, perceived burdensomeness while simultaneously increasing social support and resilience. With the limitations of a small sample size, we do not know the true effect on participants of being in a support group with other people who have also experienced suicide attempt and being able to freely talk about what has been a very heavily stigmatized part of people’s lives. Longitudinal research studies could help us explore trajectories of coping skills and improvements in functioning and recovery as a result of participating in such support groups.

## Supplementary Information

Below is the link to the electronic supplementary material.

Supplementary file1 (DOCX 12 kb)
