# Supplementary material for: A Psychoeducational Support Group Intervention for People Who Have Attempted Suicide: An Open Trial with Promising Preliminary Findings
Source: Community Ment Health J. 2022 May 30;58(8):1621–9. doi: 10.1007/s10597-022-00978-y (PMC9150625; doi:10.1007/s10597-022-00978-y)
Supplement: Supplementary file 1 — Supplementary file1 (DOCX 12 kb) [file 10597_2022_978_MOESM1_ESM.docx]

**Eclipse Psychoeducational Support Group Topics and Group Processes**

The group content was structured around a weekly topic (WT) addressing the emotional and practical needs of participants, starting with setting group rapport and safety (WT1 introductions, WT2 talking about suicide), moving on to an emphasis on tools and resources to stay safe from future suicidal ideation and attempts (WT3 giving and receiving support, WT4 what triggers suicidal thoughts, WT5 how can I cope with suicidal thoughts), and finishing with closing the group (WT6 resources, WT7 hope, WT8 where do we go from here). The program was delivered via informal discussions, where the Lifeline facilitator would guide the discussion to touch on the weekly content. Sessions also included other interactive exercises such as educational games and videos prompts. Between weekly group sessions, the peer facilitator checked in with participants via phone. These short check-ins consisted of general conversations about how the participant had been feeling since the group session. Participants could request additional check-ins.
